# The PI-3-Kinase P110α Catalytic Subunit of T Lymphocytes Modulates Collagen-Induced Arthritis

**DOI:** 10.3390/ijms22126405

**Published:** 2021-06-15

**Authors:** María Montes-Casado, Gloria Ojeda, Gabriel Criado, José M. Rojo, Pilar Portolés

**Affiliations:** 1 Centro Nacional de Microbiología, Instituto de Salud Carlos III (ISCIII), Majadahonda, 28220 Madrid, Spain; mmontes@isciii.es (M.M.-C.); gojeda@isciii.es (G.O.); 2 Grupo de Enfermedades Inflamatorias y Autoinmunes, Instituto de Investigación Hospital 12 de Octubre (i+12), 28041 Madrid, Spain; gcriado@h12o.es; 3 Centro de Investigaciones Biológicas Margarita Salas, Departamento de Biomedicina Molecular, Consejo Superior de Investigaciones Científicas (CSIC), 28040 Madrid, Spain; 4 Presidencia, Consejo Superior de Investigaciones Científicas (CSIC), 28006 Madrid, Spain

**Keywords:** arthritis, CIA, Class I phosphoinositide-3 kinase, PI3K, p110α, T cell, ICOS, signaling, CXCR5

## Abstract

The phosphatidylinositol 3-kinase (PI3K) family of enzymes plays a determinant role in inflammation and autoimmune responses. However, the implication of the different isoforms of catalytic subunits in these processes is not clear. Rheumatoid arthritis (RA) is a chronic, systemic autoimmune inflammatory disease that entails innate and adaptive immune response elements in which PI3K is a potential hub for immune modulation. In a mouse transgenic model with T-cell-specific deletion of p110α catalytic chain (p110α^−/−^ΔT), we show the modulation of collagen-induced arthritis (CIA) by this isoform of PI3K. In established arthritis, p110α^−/−^ΔT mice show decreased prevalence of illness than their control siblings, higher IgG1 titers and lower levels of IL-6 in serum, together with decreased ex vivo Collagen II (CII)-induced proliferation, IL-17A secretion and proportion of naive T cells in the lymph nodes. In a pre-arthritis phase, at 13 days post-Ag, T-cell-specific deletion of p110α chain induced an increased, less pathogenic IgG1/IgG2a antibodies ratio; changes in the fraction of naive and effector CD4^+^ subpopulations; and an increased number of CXCR5^+^ T cells in the draining lymph nodes of the p110α^−/−^ΔT mice. Strikingly, T-cell blasts in vitro obtained from non-immunized p110α^−/−^ΔT mice showed an increased expression of CXCR5, CD44 and ICOS surface markers and defective ICOS-induced signaling towards Akt phosphorylation. These results, plus the accumulation of cells in the lymph nodes in the early phase of the process, could explain the diminished illness incidence and prevalence in the p110α^−/−^ΔT mice and suggests a modulation of CIA by the p110α catalytic chain of PI3K, opening new avenues of intervention in T-cell-directed therapies to autoimmune diseases.

## 1. Introduction

Rheumatoid arthritis is a chronic, systemic autoimmune inflammatory disease that primarily affects the joints and that is characterized by progressive destruction of articular structures, causing articular pain, inflammation, stiffness and loss of function. Despite the often localized nature of the injury, the autoimmune attack is directed at elements in the connective tissue present in many organ structures, so that it is considered a diffuse connective tissue disease. Similar to many autoimmune diseases, the precise cause(s) triggering of the disease are largely unknown, yet it is known that the genetic background and some hormonal stimuli, as well as environmental and lifestyle factors, contribute to its development [1,2]. Angiogenesis, the growth of new blood vessels, is essential in the pathogenesis of RA facilitating the invasion of inflammatory cells and increase in local pain receptors that contribute to structural damage and pain [3].

As an autoimmune disease, yet unknown environmental challenges activate innate immune-response elements, providing an adjuvant signal for the induction of adaptive immune responses dependent on CD4^+^ helper T cells. This leads to the production of antibodies specific for joint self-antigens producing an inflammatory reaction [2,4]. All of these processes should be sensitive to inhibitors of steps essential to the development of adaptive immune responses, including antigen recognition and activation, or costimulatory, cytokine or chemokine signaling [4]. Class I phosphoinositide-3 kinases (PI3K) signal downstream of all of these receptor molecules, and hence PI3K is a potential hub for immune modulation. In fact, class I PI3K recruiting and activation is a hallmark of antigen recognition and of signaling by T (CD28, ICOS) and B (CD19) costimulatory molecules.

Class I PI3K are heterodimers of regulatory and catalytic subunits. Regulatory subunits are p85α, p50α, p55α, p85β and p55γ in class IA PI3K, and p101, p84 and p87 subunits in class IB PI3K. In the steady state, regulatory subunits negatively control the activity of catalytic subunits (p110α, p110β and p110δ in class IA PI3K; p110γ in class IB PI3K). Binding of regulatory subunits to phosphorylated tyrosine motifs (Class IA) or G-protein-coupled receptors (Class IB) activate the catalytic subunits to generate PI(3,4,5)P3 (PIP3) from PI(4,5)P2 in cell membranes. All subunits are ubiquitously expressed, except p110δ and p110γ, which have a preferential expression in leukocytes [5,6].

The expression of p110δ and p110γ PI3K catalytic subunits by leukocytes has focused on these subunits the research on PI3K function in immune responses. Indeed, p110δ and p110γ are functionally very important to the development of immune responses and to the function and homeostasis of immune cells, as determined by using specific inhibitors or deletion and inactivation models. In fact, inhibitors specific for p110δ, p110γ or both can attenuate rheumatoid arthritis in humans, as well as the development of arthritis in different experimental models [7,8,9,10].

However, available data from p110δ-deficient mice or humans with loss-of-function mutations of p110δ also show immune dysregulation leading to chronic intestinal inflammation in addition to immunodeficiency [11,12,13,14]. A similar intestinal inflammatory condition has been described in cancer patients under therapy with p110δ-specific inhibitors [15]. Thus, PI3K-isoform deficiency can produce chronic inflammatory conditions, at least in the case of p110δ.

Interestingly, p110α subunits are also expressed by leukocytes, and their levels are similar to those of p110δ in T lymphocytes [16]. Unlike p110δ or p110γ, p110α is essential to early fetal development due to its essential role in angiogenesis and endothelial cell migration [17]. Thus, the impact of p110α on immune responses and autoimmune diseases has to be analyzed by using either specific inhibitors, or mice with lineage-specific p110α deficiency to avoid the lethality of the animal model. PI3K p110α inhibitors can attenuate the responses of B and T lymphocytes and T-cell lines in vitro, particularly when combined with p110δ and DNA/PK inhibitors [16,18,19]. In fact, administration of a dual p110α/p110δ PI3K inhibitor inhibited antigen-specific T-cell and antibody responses in vivo, and suppressed clinical symptoms in a model of collagen-induced arthritis (CIA), as did a dual p110α/DNA-PK inhibitor in experimental autoimmune encephalomyelitis (EAE) [16,18,19].

Data on p110α function using lineage-specific deficiency are scarce. Deficiency of p110α in B lymphocytes shows a redundant role with p110δ concerning their development and survival [20]. Of interest to autoimmune diseases, the expression of constitutively active p110α allowed B cells with high affinity for self-antigens to escape deletion during differentiation and go into the periphery [21].

Our previous results from using a model of T-cell-specific deletion of p110α show that CD4^+^ and CD8^+^ T-cell activation in vitro is enhanced, with enhanced early signaling or cytokine production by Th1, Th17 and Tfh cells [22]. Production of cytokines and other effector molecules was also enhanced in p110α-deficient CD8^+^ T lymphocytes. These mice showed improved anti-melanoma responses, as shown by enhanced survival and tumor-specific cytokine responses [22]. Given the importance of antibodies to arthritis pathology, it is important to note that antibody responses to a soluble antigen were also enhanced in these deficient mice p110α [22].

Recent data show that specific removal of p110α in regulatory T (Treg) cells further lowered the impaired Treg numbers and function caused by p110δ loss [7]. In keeping with these data, Treg-specific loss of both p110α and p110δ, but not either alone increased the severity of EAE and lead to spontaneous inflammation in peripheral nerves [7]. Thus, the final impact of PI3K isoforms in immune responses, including autoimmune responses, depends on the balance of their effects on the function(s) of the different cell subpopulations involved.

Here, we have further explored the role of the p110α in T-cell dependent autoimmune responses. Using mice with a T-cell-specific loss of p110α PI3K (p110α^−/−^ΔT), we have analyzed the development of collagen-induced arthritis and collagen-specific antibody and T-cell responses. Our data show lower illness prevalence in the p110α^−/−^ΔT mice, in parallel with lower levels of IL-6 in serum and IL-17A secretion in response to collagen II in vitro. Moreover, in short-term experiments, at pre-arthritis phases, an accumulation of lymphoid cells in the lymph node of p110α^−/−^ΔT mice was found. The possible implication of a deficit in PI3K p110α-ICOS signaling axis in CIA attenuation is discussed.

## 2. Results

### 2.1. P110α^−^^/^^−^ΔT Mice Show a Lower Prevalence of CIA

PI3-kinase p110α is critically involved in angiogenesis, and thus null mice for this isoform are embryonic lethal [17]. Hence, conditional deletion models of mice have to be used to assess the tissue-specific implication of p110α in vivo. Thus, to analyze the role of p110α PI3-kinase in T-cell function in an experimental model of induced arthritis, mice with conditional deletion of the *pik3ca* gene in T cells were generated as we have previously reported [22]. Litters yield CD4-Cre^+/^^−^/p110α^flox/flox^ mice (hereinafter referred to as p110α^−^^/^^−^ΔT) and CD4-Cre^−/^^−^/p110α^flox/flox^ littermates (which will be termed wild type, WT). P110α^−^^/^^−^ΔT mice and WT littermates were injected with chicken collagen II emulsified in Freund’s complete adjuvant as indicated in the Methods. Animals were monitored for clinical arthritis symptoms in a blinded manner daily starting from 2 weeks after immunization, according to scoring criteria indicated in the Methods.

**Figure 1:**
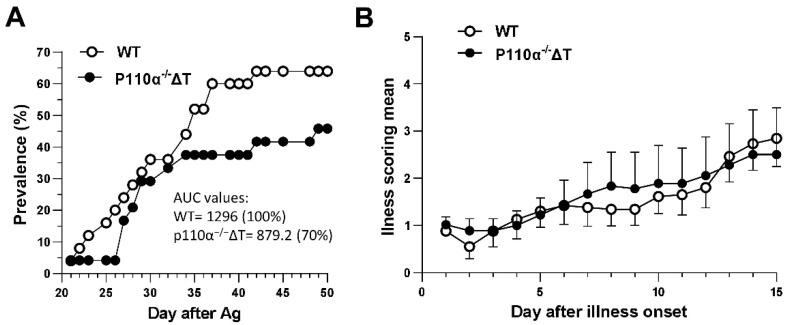
Collagen-induced arthritis in p110α^−/−^ΔT mice. Mice were immunized with type II collagen in complete Freund’s adjuvant. (**A**) Time course of prevalence of arthritis in the mice referred to day post-antigen administration. Area under the curve (AUC) values and the percent of ill mice referred to the control group are indicated. (**B**) The clinical score after the day of illness onset was recorded for 15 days. Data (mean ± SEM) from three experiments with a total *n =* 24 for control group (WT, white symbols) and *n =* 25 for p110α^−/−^ΔT mice (black symbols) are shown.

Analysis of clinical symptoms indicated a decrease in the prevalence of arthritis in p110α^−^^/^^−^ΔT mice (Figure 1A) with a reduction of the area under the curve value (AUC). A delay in the peak day of prevalence (day 49 in p110α^−^^/^^−^ΔT vs. day 42 for WT) was also observed (Figure 1A). The illness scoring of each mouse was referred to the day of illness onset and Figure 1B shows no differences between p110α^−^^/^^−^ΔT and WT groups concerning this parameter. The day of illness onset did not reach a significant difference in both groups of mice either (Supplementary Materials Figure S1A).

### 2.2. Impact of T-Cell-Specific Loss of p110α in Collagen-Specific Abs and Serum IL-6

**Figure 2:**
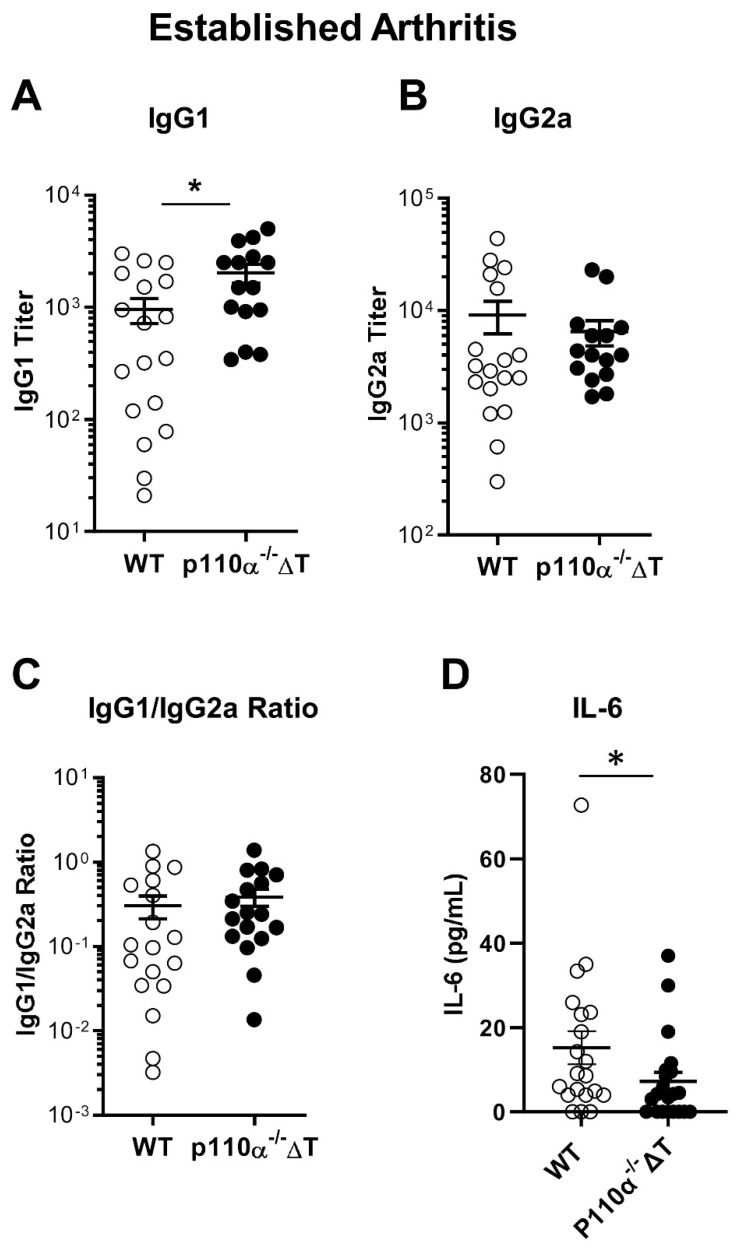
Analysis of the sera of p110α^−/−^ΔT mice under CIA. At the end of the experiment blood sera were obtained and the titer of anti-CII Abs was analyzed by ELISA. (**A**) Titer of IgG1 anti-CII antibodies in control (WT, white symbols) and p110α^−/−^ΔT (black symbols) mice. (**B**) Titer of IgG2a anti-CII antibodies. (**C**) Ratio of IgG1/IgG2a anti-CII antibody titer for each mouse. (**D**) IL-6 concentration in the sera of WT or p110α^−/−^ΔT mice was measured by ELISA. * *p* < 0.05; *n =* 21 for control group (white symbols) and *n =* 20 for p110α^−/−^ΔT mice (black symbols). Data show the mean ± SEM from individual mice analyzed.

As CIA pathogenesis is mediated by T- and B-lymphocytes, we studied both types of immune cell responses. Thus, at the end of long term experiments for established arthritis, the mice were bled, sera were obtained and analyzed for collagen-specific Abs. Figure 2A shows a higher titer of protective IgG1 anti-CIA antibodies in p110α^−^^/^^−^ΔT mice. No significant differences were found in IgG2a collagen-specific Abs between p110α^−^^/^^−^ΔT and WT groups at this stage (Figure 2B). The ratio of IgG1/IgG2a antibodies was not different in both groups either (Figure 2C). Taken together, these results suggest that the differences in CIA incidence or prevalence between p110α^−^^/^^−^ΔT and WT mice might be modulated by the abundance of protective IgG1 collagen-specific Abs.

When we analyzed IL-6 in serum, we found lower levels of this pro-inflammatory cytokine in the sera of p110α^−^^/^^−^ΔT mice than in their WT littermates (Figure 2D), which may contribute to the lower prevalence of CIA.

### 2.3. Ex Vivo Response of Lymph Node Cells in CIA in the p110α^−^^/^^−^ΔT Mouse Model

**Figure 3:**
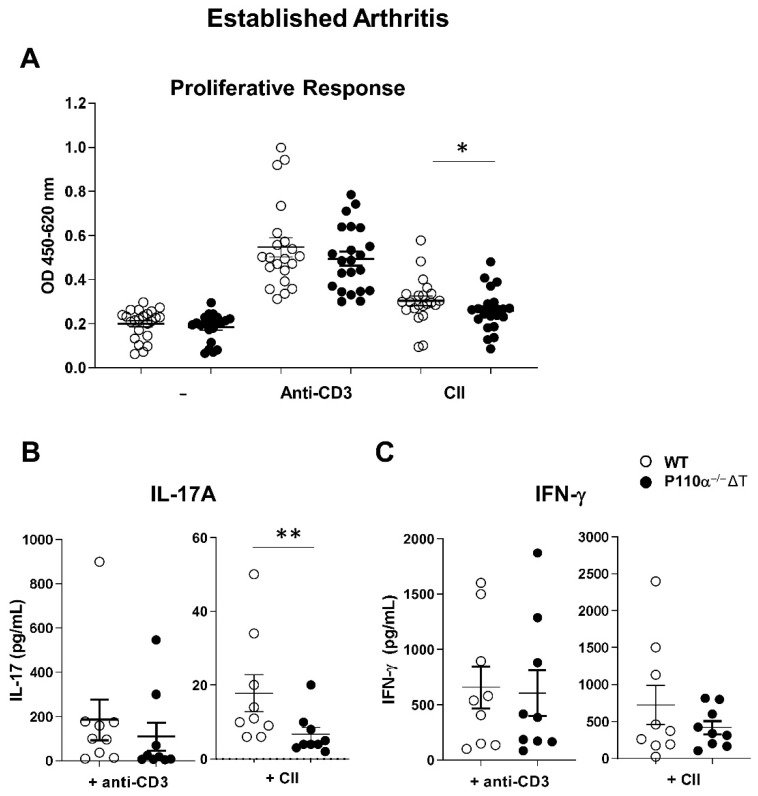
Analysis of proliferative and effector response in lymph node cells of p110α^−/−^ΔT mice under CIA (established arthritis). (**A**) The proliferative responses of lymph node cells from WT (white symbols) and p110α^−/−^ΔT (black symbols) mice were measured with WST-1 assay after three days of in vitro stimulation with anti-CD3 Ab, antigen (Collagen II, CII) or vehicle (PBS, -) as indicated in the figure legend. Mice were individually analyzed (*n =* 22 for each group) and cultures were set up in triplicates. Content of the cytokines IL-17A (**B**) and IFN-γ (**C**) in 48 h culture supernatants of lymph node cells stimulated by anti-CD3 or CII was measured by ELISA. * *p* < 0.05; ** *p* < 0.01. In (**B**,**C**), one representative experiment out of three is shown, in which *n* = 9 for WT and *n* = 9 for p110α^−/−^ΔT. Data show the mean ± SEM from individual mice analyzed.

Upon termination of long term experiment (established arthritis) the mice were sacrificed and the draining inguinal lymph nodes excised. Ex vivo stimulation of the cells was performed and proliferative response (at 72 h) and cytokine production (at 48 h) were analyzed. Proliferation of p110α^−^^/^^−^ΔT and WT lymph node cells under polyclonal (anti-CD3) stimulus or antigen (Collagen II) added to the cultures were compared. Spontaneous or anti-CD3 induced proliferative response did not show differences in p110α^−^^/^^−^ΔT vs. WT lymph node cells (Figure 3A). In contrast, collagen-specific proliferation was significantly lower in p110α^−^^/^^−^ΔT-cell cultures (Figure 3A).

To analyze the effector response of stimulated cells, 48 h culture supernatants were obtained and cytokines were evaluated by ELISA. IL-17A secreted by lymph node cells under anti-CD3 stimulation was not different in p110α^−^^/^^−^ΔT or WT cells (Figure 3B, left); however, lower levels of secreted IL-17A were obtained in collagen-stimulated p110α^−^^/^^−^ΔT cultures as compared to WT cells (Figure 3B, right). IFN-γ levels did not shown significant differences between p110α^−^^/^^−^ΔT and WT lymph node cells when they were stimulated by either anti-CD3 Ab or collagen ex vivo (Figure 3C), even though IFN-γ levels were lower in collagen-activated p110α^−^^/^^−^ΔT cells.

### 2.4. Analysis of Lymph Node Subpopulations in p110α^−^^/^^−^ΔT Mice under CIA

**Figure 4:**
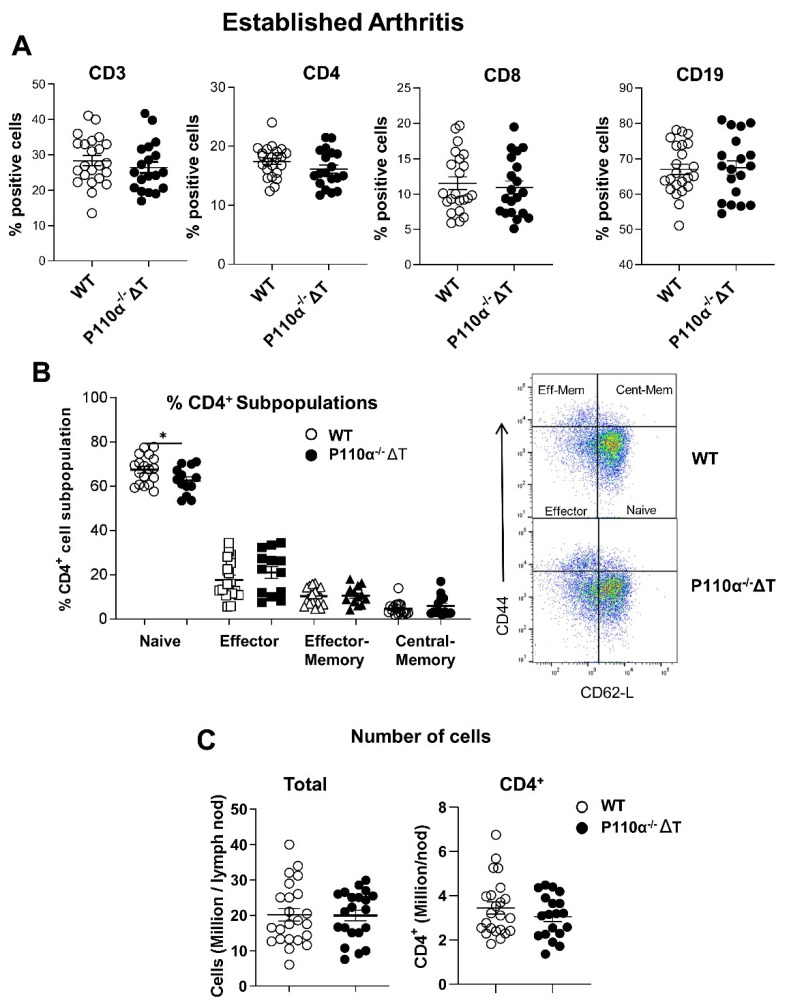
Analysis of T-cell subpopulations in the lymph nodes of p110α^−/−^ΔT mice under established CIA (long-term experiments). (**A**) Percentage of CD3^+^, CD4^+^, CD8^+^ and CD19^+^ lymphocyte subpopulations were analyzed by flow cytometry in the lymph nodes cells of WT and p110α^−/−^ΔT mice. (**B**) Percentage of naive, effector and memory CD4^+^ T cells, based on CD62-L and CD44 expression (left). Representative dot-plots showing the expression of CD44 and CD62-L in gated CD4^+^ T cells and the distribution of naive, effector and memory subpopulations are shown ((**B**), right). (**C**) The number of total (left) or CD4^+^ (right) cells per draining lymph node of WT and p110α^−/−^ΔT mice were calculated and are represented. (**A**,**C**), *n =* 23 for WT and *n =* 20 for p110α^−/−^ΔT mice; (**B**) *n =* 18 for WT and *n =* 13 for p110α^−/−^ΔT mice. * *p* < 0.05. Open symbols, WT mice; closed symbols, p110α^−/−^ΔT mice. Data show the mean ± SEM from individual mice analyzed.

We have previously shown some differences in T-cell subpopulations (i.e., decrease in CD4^+^ T-cell number) in p110α^−^^/^^−^ΔT in homeostasis [22]; however, the balance of cell subpopulations in a long term experimental model as CIA had not been studied. Thus, we aimed to analyze the cell subpopulation composition in the draining lymph nodes of p110α^−^^/^^−^ΔT mice in the CIA model, in which T and B cell are implicated. No differences between p110α^−^^/^^−^ΔT and WT were found in terms of whole CD3^+^, CD8^+^ or CD19^+^ cell subpopulations (Figure 4A) in established arthritis. Although the fraction of CD4^+^ cells showed a slight decrease in p110α^−^^/^^−^ΔT under CIA, the difference was not statistically significant (Figure 4A). Then we analyzed the different subpopulations of CD4^+^ T cells based on CD44 and CD62-L expression, namely naive, effector and memory T-cell subpopulations. Figure 4B shows a decrease in p110α^−^^/^^−^ΔT naive CD4^+^ T cells without significant changes in other CD4^+^ T-cell subpopulations. No differences were observed between p110α^−^^/^^−^ΔT and WT mice in the total number of cells or in the number of CD4^+^ cells in the lymph nodes (Figure 4C). Accordingly, numbers of naive CD4^+^ cells were also diminished in the p110α^−^^/^^−^ΔT mice (Supplementary Materials Figure S1B).

### 2.5. Analysis of the Immune Response to Collagen II in p110α^−/−^ΔT Mice before the Arthritis Onset

**Figure 5:**
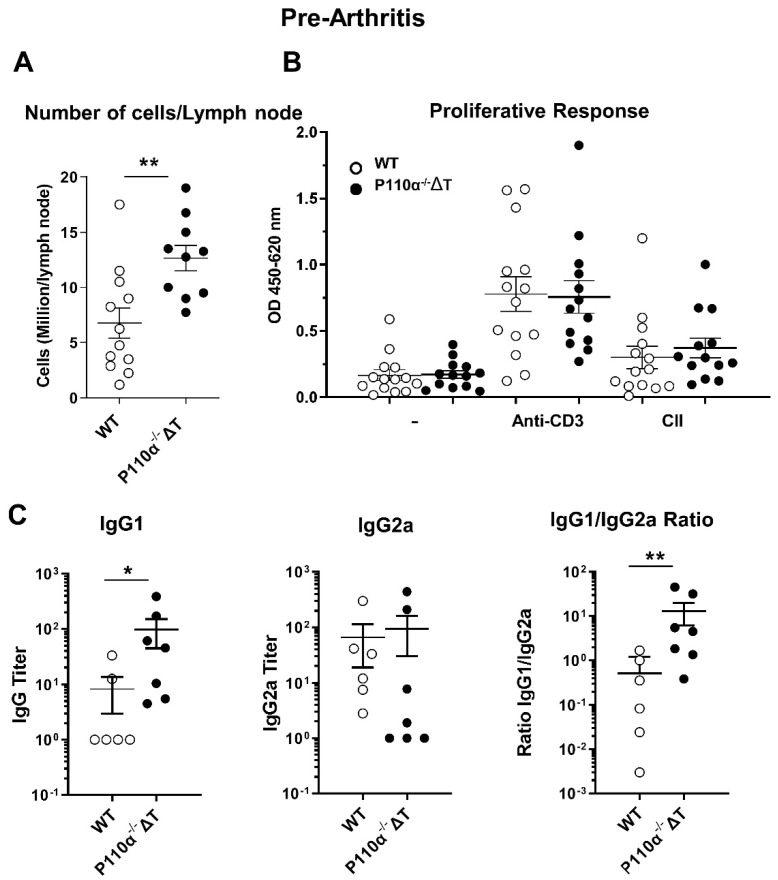
Immune response to Collagen II in p110α^−/−^ΔT mice before the onset of arthritis. Thirteen days after CII in CFA injection, WT and p110α^−/−^ΔT mice were analyzed. (**A**) Number of cells per draining lymph node. (**B**) The proliferative responses of lymph node cells from WT (white symbols) and p110α^−/−^ΔT (black symbols) mice were measured with WST-1 assay, after three days of in vitro stimulation with anti-CD3 Ab, Collagen II (CII) or vehicle (PBS, -) as indicated in the figure. Mice were individually analyzed (*n =* 14 for WT and *n =* 13 for p110α^−/−^ΔT) and cultures were set up in triplicates. (**C**) The level of IgG1 (**C**, left) and IgG2a ((**C**), center) anti-CII Abs in the sera was analyzed by ELISA in WT (white symbols) and p110α^−/−^ΔT (black symbols) mice. The ratio of anti-CII IgG1/IgG2a antibody titer for each mouse in a representative experiment is shown in (**C**), (right panel). * *p* < 0.05; ** *p* < 0.01. Data show the mean ± SEM from individual mice analyzed.

To further understand the early processes which determine the attenuated development of CIA in the p110α^−/−^ΔT, we set up experiments to analyze the immune response to CII at a shorter time, namely 13 days post-Ag-immunization, in a pre-arthritis stage. When the draining lymph nodes of the mice were excised, an increased cell number was observed in p110α^−/−^ΔT mice (Figure 5A). This higher cell content is not related to an increased proliferative response to CII in the lymph node cells, as lymph node cells from WT or p110α^−/−^ΔT mice do not show significant differences in proliferation when activated with either anti-CD3 Ab or CII ex vivo (Figure 5B).

Although the mice did not show clinical symptoms of arthritis at this time, the sera collected at 13 days post-Ag were analyzed for collagen-specific Abs. At this stage we found an increased content of IgG1 anti-CII Abs in the sera of p110α^−^^/^^−^ΔT mice (Figure 5C, left) and no differences in anti-CII IgG2a. The ratio IgG1/IgG2a was significantly higher in p110α^−/−^ΔT mice, suggesting a more favorable balance in protective vs. pathogenic isotypes for these animals; this could favor the latter decrease in prevalence of CIA found at longer times (Figure 1).

**Figure 6:**
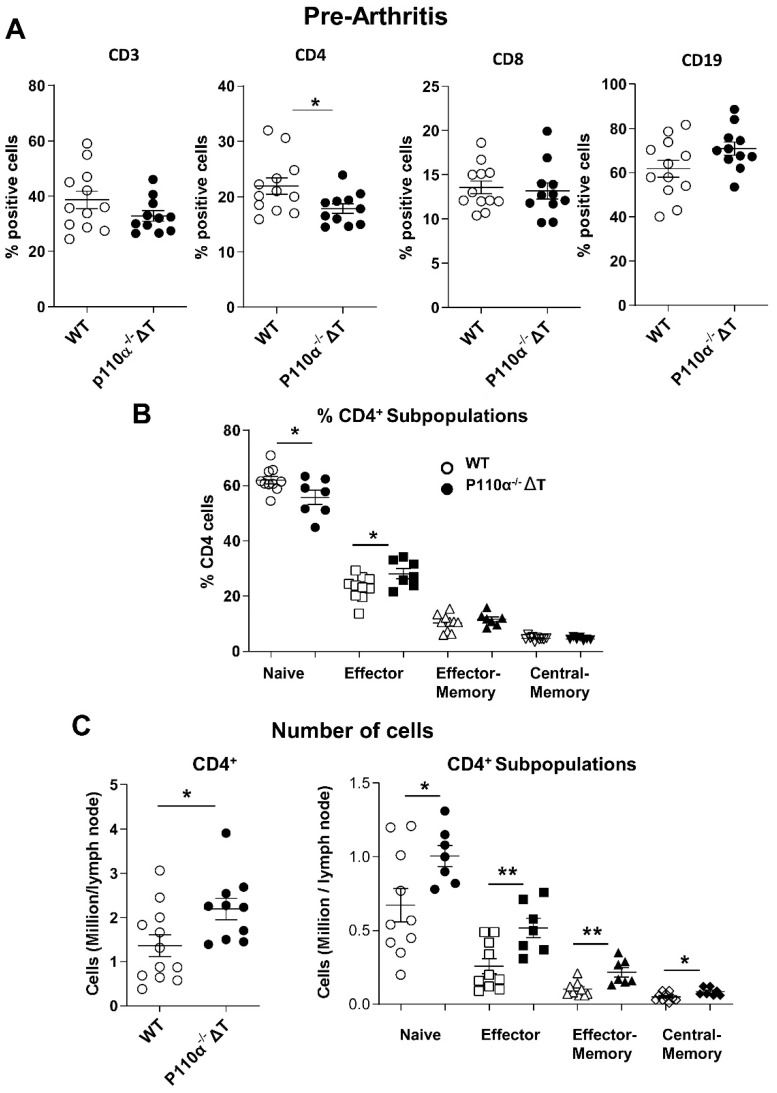
Analysis of T-cell subpopulations in the lymph nodes of p110α^−/−^ΔT mice before the onset of arthritis (13 days post-Ag administration). (**A**) Percentage of CD3^+^, CD4^+^, CD8^+^ and CD19^+^ lymphocytes in the draining lymph nodes of WT and p110α^−/−^ΔT mice, as indicated. (**B**) Fraction of naive, effector and memory CD4^+^ T cells, based on CD62-L and CD44 expression. (**C**) The number of CD4^+^ cells (millions per draining lymph node, left) or the number of naive/effector/memory CD4^+^ cells per lymph node (right) in immunized WT and p110α^−/−^ΔT mice were determined. Mice were individually analyzed; *n =* 12 for WT and *n =* 10 for p110α^−/−^ΔT mice. For naive/effector/memory subpopulation analysis, n was 10 for WT and 7 for p110α^−/−^ΔT mice. Open symbols, WT mice; closed symbols, p110α^−/−^ΔT mice. * *p* < 0.05; ** *p* < 0.01. Data show the mean ± SEM from individual mice.

At this stage of 13 days post-CII immunization, when clinical symptoms are not yet visible, we observed a lower percentage of CD4^+^ cells in p110α^−^^/^^−^ΔT mice (Figure 6A), as previously reported under steady-state conditions [22]. No significant changes in the percentage of CD8^+^ or CD19^+^ cells were observed (Figure 6A). The fraction of naive CD4^+^ cells showed a decrease while the fraction of effector subpopulation was increased in p110α^−^^/^^−^ΔT (Figure 6B). However, in accordance to the higher cell number per draining lymph node in p110α^−^^/^^−^ΔT mice (Figure 5A), the absolute cell numbers of all CD4^+^ subpopulations analyzed were higher in the p110α^−^^/^^−^ΔT mice (Figure 6C). These included not only naive effector or memory cells but also T-regulatory CD4^+^CD25^+^FoxP3^+^ cells (Supplementary Materials Figure S1C) and CD8^+^ cells (Supplementary Materials Figure S2C).

As CD8^+^ T cells play a significant role in the pathogenic immune response in rheumatoid arthritis [23,24,25], and CD8^+^ T cells are also affected by the deletion of p110α catalytic subunit of PI3K [22], they were also analyzed. The subpopulation composition of CD8^+^ cells in the draining lymph nodes of p110α^−^^/^^−^ΔT mice in the established CIA (long term experiments) was determined. As in the case of CD4^+^ T cells (Figure 4), the percentage of total CD8^+^ cells was not affected in the p110α^−^^/^^−^ΔT mice; however, the fraction of naive CD8^+^ subpopulation was diminished in these animals in the CIA model (Supplementary Materials Figure S2A). When short term (13 days) CII immunization experiments were performed, in pre-arthritis stage, we did not find differences in the fractions of total CD8^+^ (Figure 6A) or in CD8^+^ T-cells subpopulations in p110α^−^^/^^−^ΔT mice (Supplementary Materials Figure S2B), making a difference with CD4^+^ T cells (Figure 6); but when cell numbers in the lymph node were considered, we found a clear increase of CD8^+^ subpopulations in the animals bearing the genetic modification of p110α PI3K (Supplementary Materials Figure S2C), as was observed in CD4^+^ cells.

### 2.6. Effect of Genetic Deletion of p110α in Homing Markers Expression and ICOS-Dependent T-Cell Signaling

**Figure 7:**
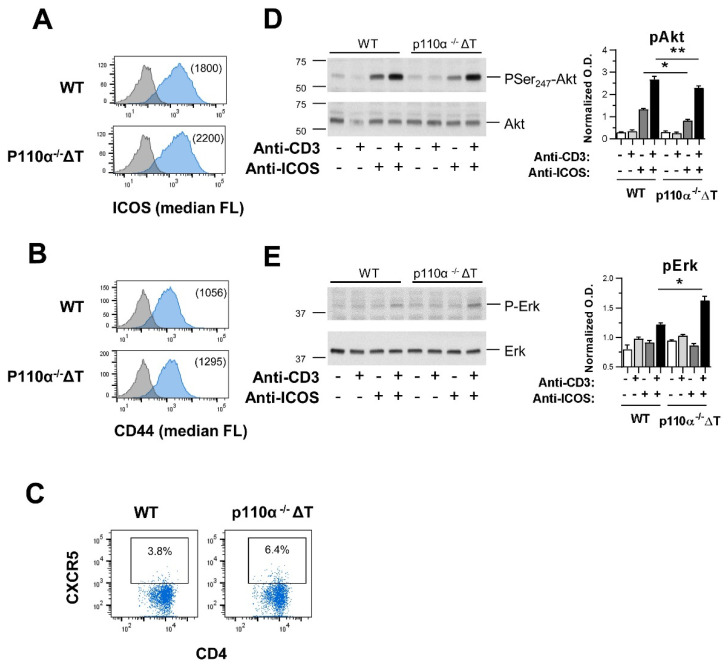
In vitro analysis of T-cell blasts of p110α^−/−^ΔT: expression of surface markers and intracellular signaling. Differences in the expression of (**A**) ICOS, (**B**) CD44, or (**C**) CXCR5 in CD4^+^ T-cell blasts from WT and p110α^−/−^ΔT mice. Numbers in parenthesis in (**A**,**B**) are the median of fluorescence intensity for ICOS and CD44, respectively. The percentage of CXCR5^+^ cells in (**C**) is indicated in each figure. (**D**,**E**) Early intracellular signaling measured as (**D**) phospho-Akt_Ser473_ and (**E**) phospho-Erk in CD4^+^ T-cell blasts activated for 20 min with latex beads coated with anti-CD3, anti-ICOS, anti-CD3 and anti-ICOS antibodies, or control antibodies, as indicated. Phospho-Akt_Ser473_ (**D**) or phospho-Erk (**E**) in the cell lysates was determined by immunoblot with specific antibodies. Protein load was determined with anti-Akt or anti-Erk antibodies to determine normalized O.D. values, as shown in accompanying graphs (right). Graphs show mean ± SEM of triplicate determinations. * *p* < 0.05; ** *p* < 0.01.

We considered whether the higher number of cells observed in p110α^−^^/^^−^ΔT mice in pre-arthritis stage could be due to an increased expression of homing surface markers, such as CXCR5 and CD44, in activated T cells of p110α^−^^/^^−^ΔT mice. Since ICOS signaling through PI3K is essential to the development of CIA [26,27], ICOS expression and signaling were also studied. Thus, blasts were obtained by activation of CD4^+^ T cells from unimmunized WT and p110α^−^^/^^−^ΔT mice, and expression of different markers was determined. After four days in culture, the cells from p110α^−^^/^^−^ΔT mice showed an increased expression of ICOS, CD44 and CXCR5 (a migration regulator marker characteristic of T follicular helper CD4^+^ cells) as compared to WT T-cell blasts (Figure 7A–C). When intracellular signaling was analyzed we found a decrease in the PI3K-dependent phosphorylation of Akt induced by ICOS alone or when ICOS acts as a TCR-co-stimulus in the p110α^−^^/^^−^ΔT blasts (Figure 7D). However, Erk-phosphorylation was enhanced in the same cells (Figure 7E), in agreement with previous data [16,22]. These results indicate that genetic deletion of p110α produce altered ICOS-mediated signaling concomitant with up-regulation of the expression of ICOS and homing molecules, as CXCR5 and CD44, in CD4^+^ T-cell blasts.

**Figure 8:**
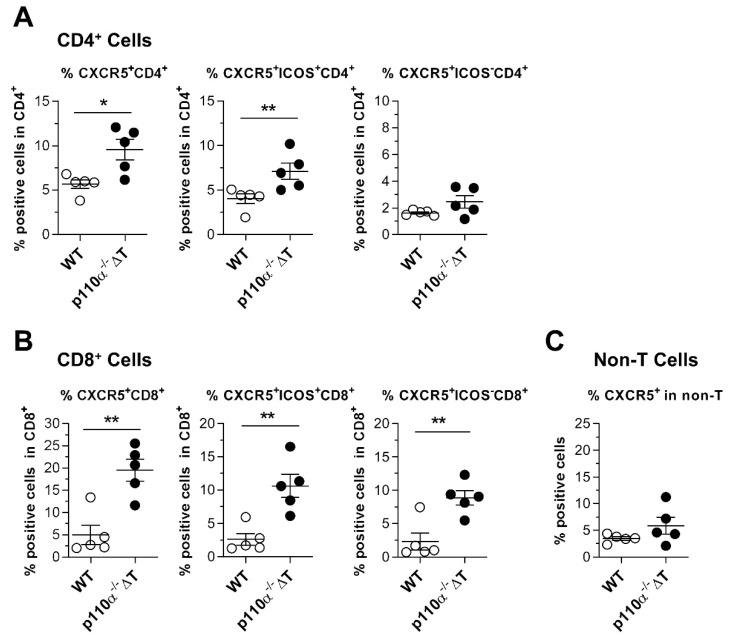
Enhanced expression of the migration regulator CXCR5 in T cells from the draining lymph nodes of p110α^−/−^ΔT mice after 13 days of Collagen-II-immunization. (**A**) Expression of CXCR5 in gated CD4^+^ T cells, as percentage of CXCR5^+^CD4^+^ T cells (left panel), CXCR5^+^ICOS^+^ CD4^+^ T cells (center panel) or CXCR5^+^ICOS^−^ CD4^+^ T cells (right panel). (**B**) Same as in (**A**), for gated CD8^+^ T lymphocytes. (**C**) Percentage of CXCR5^+^ cells in CD4^−^CD8^−^ lymph node cells (non-T cells). Data show the mean ± SEM from individual mice (*n =* 5 per group). * *p* < 0.05; ** *p* < 0.01.

In view of the former results, we considered the possibility that increased T-cell homing was retaining the cells in draining lymph nodes of p110α^−^^/^^−^ΔT mice under CII-immunization and we aimed to analyze the cell surface expression of CXCR5. Figure 8 shows an increase in CXCR5^+^ICOS^+^ T cells in the draining lymph nodes of p110α^−^^/^^−^ΔT mice at 13 days post-Ag. The increased CXCR5 expression is linked to the T-cell lineage, which is the target of the p110α genetic modification. Thus, CD4^+^ (Figure 8A) as well as CD8^+^ (Figure 8B) T cells show increased CXCR5 expression, but this was not observed in other lymph node cells (non-T-cell population, Figure 8C).

## 3. Discussion

PI3Ks are essential to diverse basic cell processes and are modulated by the expression pattern of PI3K isoforms, their substrates and the connections among different downstream signals [28]. However, the implication of different PI3K catalytic isoforms in T- and B-cell mediated autoimmune processes is not clear. The ubiquitous expression and the fundamental role of PI3K p110α catalytic subunit in the angiogenesis of embryos [17] makes necessary the development of animal models with lineage-specific modification of this isoform to study its role in physiology and pathology in the adult. Here, we have analyzed the effect of the T-cell-specific p110α deletion in the development of collagen-induced arthritis as a model where antibodies have a major role in the development of the disease. We show decreased prevalence of arthritis in p110α^−/−^ΔT mice, with decreased IL-6 and IL-17A secretion and enhanced anti-CII IgG1 antibodies in response to CII. In fact, our previous data from using the KLH protein showed enhanced KLH-specific IgG1 and IgG2b antibodies in the sera of immunized p110α^−/−^ΔT mice, and enhanced Tfh in their spleens [22].

The results observed in the CIA model may be based on changes that occurred in the induction phase of the disease and, given the importance of the co-stimulator ICOS in collagen-induced arthritis [26,27,29], could be partially due to the defective ICOS-mediated PI3K signaling in our p110α^−/−^ΔT mice (Figure 7).

ICOS is a key co-stimulator in the differentiation and function of follicular helper T cells and inflammatory T cells. ICOS/ICOS-ligand interactions contribute to the development of CD4^+^CXCR5^+^ Tfh, the formation of germinal centers in the lymph nodes and the production of T cell-dependent Abs [30,31]. ICOS-dependent PI3K signaling controls the induction and maintenance of collagen-induced arthritis in the mouse [27], and ICOS knockout mice on the DBA/1 background are completely resistant to collagen-induced arthritis and show no inflammation in the joints [29]. Moreover, rheumatoid arthritis patients have higher levels of CD4^+^PD-1^+^CXCR5^+^ Tfh cells in peripheral blood than healthy controls, and the frequency of CD4^+^ICOS^+^CXCR5^+^ Tfh cells is linked with DBA/1 mice undergoing CIA [32]. In CD4^+^ T lymphocytes, we have shown a preferential binding of PI3K p110α-containing complexes to phosphorylated ICOS and a role for p110α in the regulation of T cell responses in vitro and in vivo [16,18,19,22]. In accordance with this, we show here a defective ICOS-mediated signaling, with decreased Akt phosphorylation in p110α^−/−^ΔT CD4^+^ blasts (Figure 7), and a lower in vitro proliferative response to CII in p110α^−/−^ΔT lymph node cells under established CIA (Figure 3), conditions which may diminish the prevalence of CIA in these mice.

When the impact of p110α deletion in the induction phase of CIA was analyzed at 13 days post-Ag, we found an accumulation of p110α^−/−^ΔT cells in the lymph nodes of CII-immunized mice. As there were no changes in the proliferative response of p110α^−/−^ΔT cells to CII at this stage, this could be explained by a retention of the cells and increased homing capacity in the mouse p110α^−/−^ΔT. In this sense, we observed a higher proportion of CXCR5^+^ T cells in the lymph nodes of p110α^−/−^ΔT in the pre-arthritis stage (Figure 8) and an increased expression of the surface markers ICOS, CXCR5 and CD44 in T-cell blasts of p110α^−/−^ΔT mice (Figure 7). In this scenario, the possible link of ICOS with p110α PI3K and cell homing/migration is highlighted by the fact that ICOS interaction with ICOS-ligand expressed by bystander B cells is needed for Tfh cells to scroll and reach germinal centers [33,34]. Ligation of ICOS induces actin polymerization with cell elongation and generation of lamellipodia and filopodia in T cells. This phenomenon is dependent on PI3K activity, so that ICOS-induced, PI3K-dependent elongation of ICOS^+^ T cells might be involved in germinal center formation in vivo [34,35]. We have previously shown that, depending on the system, ICOS-induced elongation is mainly [16] or partially [22] dependent on the activity of the p110α PI3K subunits. Thus, one could hypothesize that the increased cell number in lymph nodes of p110α^−^^/^^−^ΔT mice may be due to enhanced homing and retention of the cells which might delay the traffic of pathogenic cells to the target tissue in the paws, influencing the changes in clinical parameters of CIA found in the p110α^−^^/^^−^ΔT model (Figure 1).

Additionally, the multifunction protein osteopontin can interact with the regulatory p85α subunit of PI3K, favoring ICOS-mediated Tfh differentiation and IgG1 production [35]. Thus, one could suggest the possibility that the loss of p110α facilitates p85α–osteopontin interactions, enhancing CXCR5 expression and IgG1 responses.

CD8^+^ T cells are also affected by the p110α-deletion in this model, and we have found an increased fraction of cells expressing the migration regulator marker CXCR5^+^ in CD8^+^ T lymphocytes as well as in CD4^+^ T cells. This enhancement was not observed in non-T cells in the p110α^−^^/^^−^ΔT mice. In accordance with the increase in CD4^+^CXCR5^+^ Tfh and CD8^+^CXCR5^+^ cells, in the short-term experiments, we detected a higher anti-CII IgG1 Ab content in the p110α^−/−^ΔT mice, and a ratio of IgG1/IgG2a balanced towards a protective isotype in the p110α^−/−^ΔT mice and towards a pathologic isotype in the WT mice (Figure 5). Interestingly, CD8^+^CXCR5^+^ T cells are a recently described subpopulation that seem to act as helper cells favoring the production of IgG1 isotype antibodies [36,37]. This condition together with the lower proliferative and effector response to CII found in the long-term experiments may favor the lower prevalence of CIA found in p110α^−^^/^^−^ΔT mice.

The implication of the PI3K/Akt/mTOR axis in immune responses and inflammation opens the door to the study of specific isoform inhibitors with therapeutic application to different pathologies. PI3K activation, however, has both positive and negative roles in immune responses, so that PI3K suppression can attenuate immune responses but can also disrupt peripheral tolerance and promote autoimmunity [38]. Although many different therapies have been used to treat autoimmune diseases, there are currently no curative therapies and it appears that a combination of approaches may be needed to provide effective and long-term protection from disease progression. Catalytic isoforms of PI3K p110δ and p110α contribute to autoimmunity [16,18,19,21,39,40,41]. The ubiquitous expression of p110α or the high expression of p110δ in T and B lymphocytes make it difficult to achieve clear conclusions on the impact of systemic treatments to autoimmune diseases without strongly interfering with normal immune responses. Our study in the p110α^−^^/^^−^ΔT model restricts the results to the effect of p110α deletion in T cells in an arthritis model and suggests new avenues of therapeutic approaches directed to target specific cell lineages by means of nanomolecules.

## 4. Materials and Methods

### 4.1. Mice

Mice used in this study were CD4-Cre [42] (strain B6;D2-Tg(Cd4-cre) 1Cwi/CwiCnrm) from the European Mouse Mutant Archive (EMMA) and *Pik3caflox*, p110α^flox/flox^ [17] from stock purchased from Charles River, all in a C57BL/6J background. They were bred under specific pathogen-free conditions in the animal care facility of the Instituto de Salud Carlos III (Majadahonda, Madrid, Spain) and used at 8-12 weeks of age at the start of the experiments. These mice were crossed to obtain CD4-Cre^−/−^ p110α^flox/flox^ (WT) and CD4-Cre^+/−^ p110α^flox/flox^ (p110α^−/−^∆T) mice, as described in Reference [22]. All experimental procedures were performed according to established institutional and national guidelines under project licenses PROEX 114/14 and PROEX 330/15 (to PP, ISCIII) approved by the Ethics and Animal Welfare Committees of the Instituto de Salud Carlos III (OEBA-Majadahonda and CEIYBA) and the Consejeria de Medio Ambiente y Ordenación del Territorio de la Comunidad de Madrid.

Mice were genotyped for Cre and floxed p110α, using the oligonucleotides and conditions previously described [17].

### 4.2. Collagen-Induced Arthritis: Induction and Assessment

WT and p110α^−/−^∆T mice were immunized intradermally, under anesthesia, at the base of the tail with an emulsion of chicken type II collagen in Freund’s complete adjuvant (200 µg per mouse) essentially as previously described [43]. Clinical evaluation of arthritis was assessed daily by scoring each limb of the mice according to the following criteria: (0) normal, (1) slight swelling and erythema, (2) pronounced edematous swelling and (3) stiffness of the joint. The degree of joint swelling for each paw (scored from 0 to 3) was assessed and expressed as the cumulative arthritis severity score for 4 paws, with a maximum possible clinical score of 12 per mouse. Scoring began 2 weeks after immunization. Arthritis onset (the first day in which clinical signs of arthritis were observed) appeared at 3–6 weeks after immunization.

In the long-term experiments (established arthritis), animals were clinically evaluated up to 60 days after collagen II administration and then euthanized before obtaining biological samples. In short-term experiments, mice were sacrificed 13 days after collagen immunization.

### 4.3. Analysis of T-Cell Responses

T-cell responses were determined as previously described [43]. Briefly, draining (inguinal) lymph nodes were removed postmortem from collagen-immunized mice. Single cell suspensions were prepared in culture medium (RPMI 1640 supplemented with L-Glutamine, Penicillin–Streptomycin, 50 µM 2-Mercaptoethanol and 10% heat-inactivated FBS) and passed through 30 µm filters to remove clumps. After washing three times with medium, the cells were counted and suspended in culture medium to dispense 2 × 10^5^ cells/well. Cell suspensions were plated in triplicates in U-bottom 96-well Falcon plates containing the appropriate stimuli: anti-CD3 Ab (5 µg/mL, YCD3-1 Ab [44]) or heat-denatured chicken CII (50 µg/mL). Same volume of PBS was used as a control in a total final culture volume of 200 µL/well.

After culturing for 48 h at 37 °C in 5% CO_2_, 100 µL supernatant/well were removed to evaluate cytokines and the rest of the cell culture was incubated for further 24 h. To determine the rate of cell proliferation, 10 µL of Cell Proliferation Reagent WST-1 (Sigma, Merck KGaA, Darmstadt, Germany) was added to each well and incubated for 2 h. Then, the optical density (O.D.) was measured at 450–620 nm in a Multiskan FC microplate photometer (Thermo Fisher Scientific, Waltham, MA, USA).

### 4.4. Measurement of Anti-Collagen Antibodies

Measurement of anti-CII Abs in the serum was performed by ELISA according to a previously described procedure [43]. Secondary horseradish peroxidase coupled anti-mouse IgG1- and anti-mouse IgG2 antibodies were from BD Biosciences (San Jose, CA, USA). Optical density was read in a Multiskan FC microplate photometer at 450–540 nm.

### 4.5. Analysis of Cytokine Production

Cytokines in culture supernatants (IL-17A and IFN-γ) and in sera (IL-6) were determined with Ready-SET-Go!^®^ kits (eBioscience, San Diego, CA, USA), according to the manufacturer’s instructions.

### 4.6. Analysis of Surface and Intracellular Markers by Flow Cytometry

Single cell suspensions were incubated for 10 min with heat-inactivated normal mouse serum in staining buffer (10% in PBS with 5% heat-inactivated FBS, 0.05% sodium azide, staining buffer) to block Fc receptors and then with the indicated labelled antibodies to detect cell-surface molecules; after extensive washing they were analyzed by flow cytometry gating lymphoid cells according to FSC/SSC. Fluorochrome-coupled antibodies against the surface antigens CD3, CD4, CD8, CD19, CD25, CD44, CD62L, CXCR5, ICOS and their appropriate isotype controls were purchased from eBioscience (San Diego, CA, USA) or BioLegend (San Diego, CA, USA). Data were acquired in a FACSCanto (BD Biosciences, San Jose, CA, USA) or in a FACS LSR Fortessa (BD Biosciences) flow cytometer and analyzed by using FACSDivaTM v 8.0.1 (BD Biosciences) or FlowJo software (Tree Star, Inc., Ashland, OR, USA, Version 10.0).

CD4^+^CD25^+^FoxP3^+^ T-regulatory cells were determined by flow cytometry. Cells were first surface stained with anti-CD4 and anti-CD25 as above and then they were suspended, washed, fixed and permeabilized with the Transcription Factor Staining Buffer Set (eBioscience). Then, the cells were stained with anti FoxP3-PE (3G3, eBioscience) or control isotype antibody according to the protocol for intracellular staining of transcription factors. Data were then acquired on a FACSCanto flow cytometer and analyzed by using FACSDivaTM or FlowJo software, as above.

### 4.7. CD4^+^ T-Cell Blasts, Generation, Activation and Western Blot

CD4^+^ T cells were isolated from the spleen of WT or p110α^−/−^ΔT mice, using the CD4^+^ T-cell isolation kit II (130-090-860, Miltenyi Biotech, Bergisch Gladbach, Germany). The cells were activated for 96 h at 10^6^ cells/mL in culture medium (Click’s medium supplemented with 10% heat inactivated fetal bovine serum) with 2.5 µg/mL Concanavalin A in the presence of 5 × 10^5^ cells/mL of Mitomycin C-treated T-cell-depleted spleen cells [19] plus human recombinant IL-2 (2 ng/mL, Peprotech EC, Ltd. (London, UK)) and mouse recombinant IL-4 (10 ng/mL, Peprotech EC, Ltd.). Then, the cells were thoroughly washed before staining for flow cytometry analysis or used in activation assays.

Phosphorylated proteins in cell lysates were determined in four day CD4^+^ blasts as described previously [19,45]. Cells were washed and activated at 37 °C for 20 min at 4 × 10^7^ cells/mL with latex beads pre-coated with antibodies (anti-CD3, clone 500A2, 0.5 µg/mL) plus anti-ICOS Ab (clone C398.4A, [46], 10 µg/mL) or control antibodies (10 µg/mL). After washing with ice-cold PBS, 500 µM EDTA and 200 µM NaVO_4_, the cells were lysed (4 × 10^7^ cells/mL in 1% Triton X-100, 50 mM Tris/HCl, 150 mM NaCl, pH 7.6, plus 1 mM MgCl2, 1 mM EGTA, 1 mM NaVO_4_ and protease inhibitor cocktail (Selleckchem, Houston, TX, USA). After 15 min on ice, the lysates were centrifuged, and the post-nuclear lysates were mixed with 4× reducing SDS Laemmli sample buffer. Separation of proteins by SDS–PAGE and immunoblot with anti-phosphoSer^473^Akt (Cell Signaling Technology, Danvers, Massachusetts, #4060), anti-phospho-ERK (V6671, Promega, Madison, WI, USA), anti-Akt and anti-Erk antibodies (Upstate Biotechnology, Inc., Lake Placid, New York, USA) were performed as described [19,45].

### 4.8. Statistical Analyses

Data were analyzed by using the GraphPad Prism 9 software (GraphPad Prism Software Inc., La Jolla, CA, USA), and they are shown as the mean ± standard error of the mean (SEM). Data from biological replicates (mice) were analyzed individually. Direct group–group comparisons were assessed by using Student’s *t*-test or Mann–Whitney test for unpaired, two-tailed experimental design. Significant differences between data are indicated by asterisks (* *p* < 0.05, ** *p* < 0.01 and *** *p* < 0.001). All statistical calculations are referred to the control group or as indicated by brackets.

## Data Availability

The data presented in this study are available from the corresponding authors on reasonable request.

## Supplementary Materials

The following are available online at https://www.mdpi.com/article/10.3390/ijms22126405/s1.
