# Supplementary material for: The PI-3-Kinase P110α Catalytic Subunit of T Lymphocytes Modulates Collagen-Induced Arthritis
Source: Int J Mol Sci. 2021 Jun 15;22(12):6405. doi: 10.3390/ijms22126405 (PMC8232790; doi:10.3390/ijms22126405)
Supplement: Supplementary file 1 [file ijms-22-06405-s001.zip › ijms-1248913-supplementary.pdf]

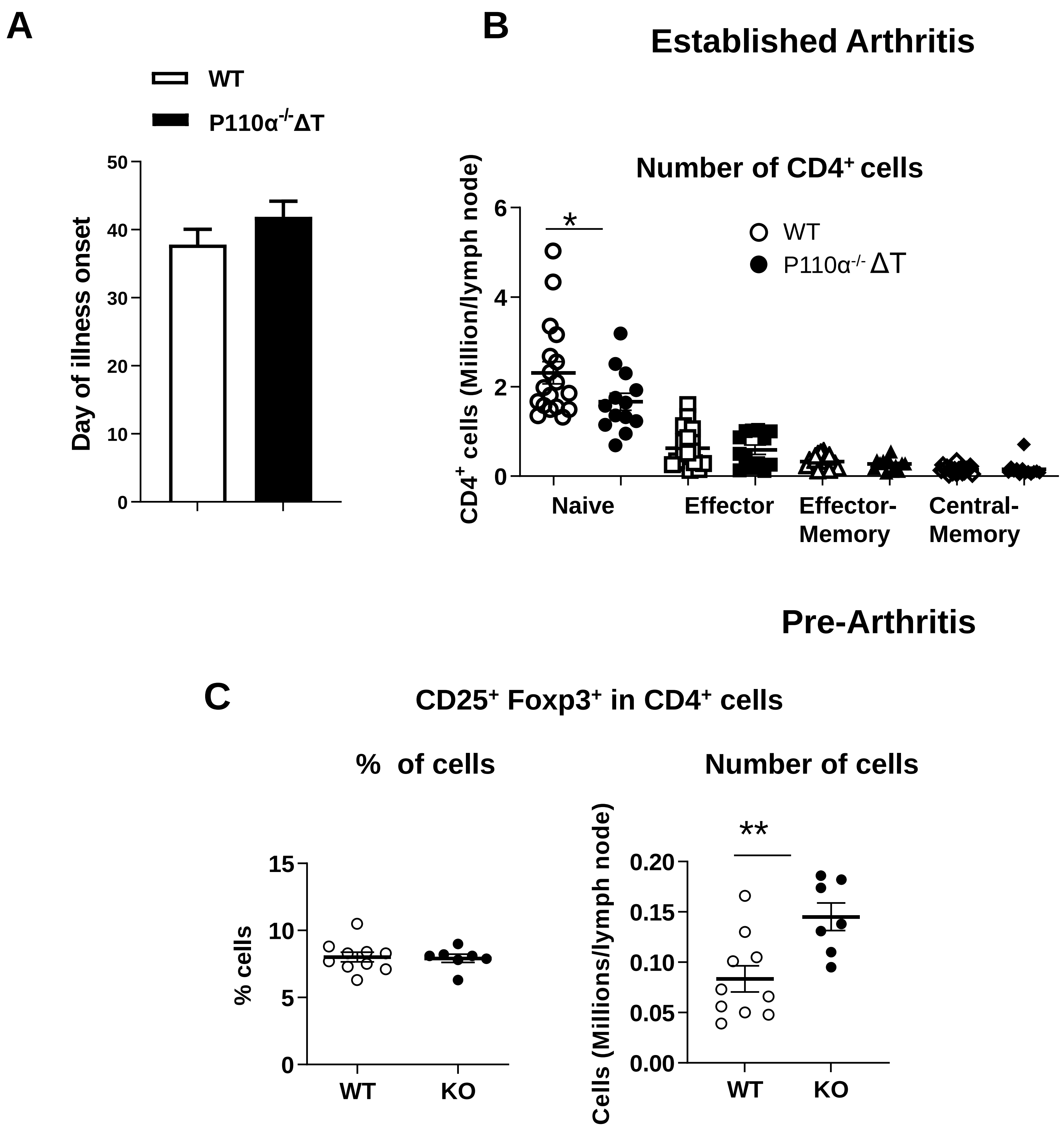

Figure S1. Collagen-Induced Arthritis in P110$\alpha^{-/-}$ ΔT mice supplementary material

A. The first day of illness symptoms (day of illness onset) after immunization of the mice with type II collagen in complete Freund's adjuvant was registered. Data are Mean±SEM. 37.80±2.25 for WT vs. 41.88±2.3 for p110$\alpha^{-/-}$ΔT. N=24 for control group (white) and n= 25 for p110$\alpha^{-/-}$ΔT mice (black).

B. Upon termination of long term experiments (established arthritis) $CD4^{+}$ cell subpopulations were analyzed by cytometry in the lymph nodes cells of WT and p110$\alpha^{-/-}$ΔT mice. Absolute numbers of naive, effector and memory $CD4^{+}$ T cells, basing in CD62-L and CD44 surface markers expression. N= 18 for WT and n=13 for p110$\alpha^{-/-}$ΔT mice. *, $p<0.05$. Data (mean ± SEM) from individual mice analyzed.

C. Thirteen days after Ag immunization (pre-arthritis) $CD4^{+}$ cell subpopulations were analyzed by cytometry in the lymph nodes cells of WT and p110$\alpha^{-/-}$ΔT mice. Percentage and absolute numbers of T regulatory CD25+Foxp3+ cells are shown, using CD25 and FoxP3 as expression markers. **, $p<0.01$. Data (mean ± SEM) from individual mice analyzed.

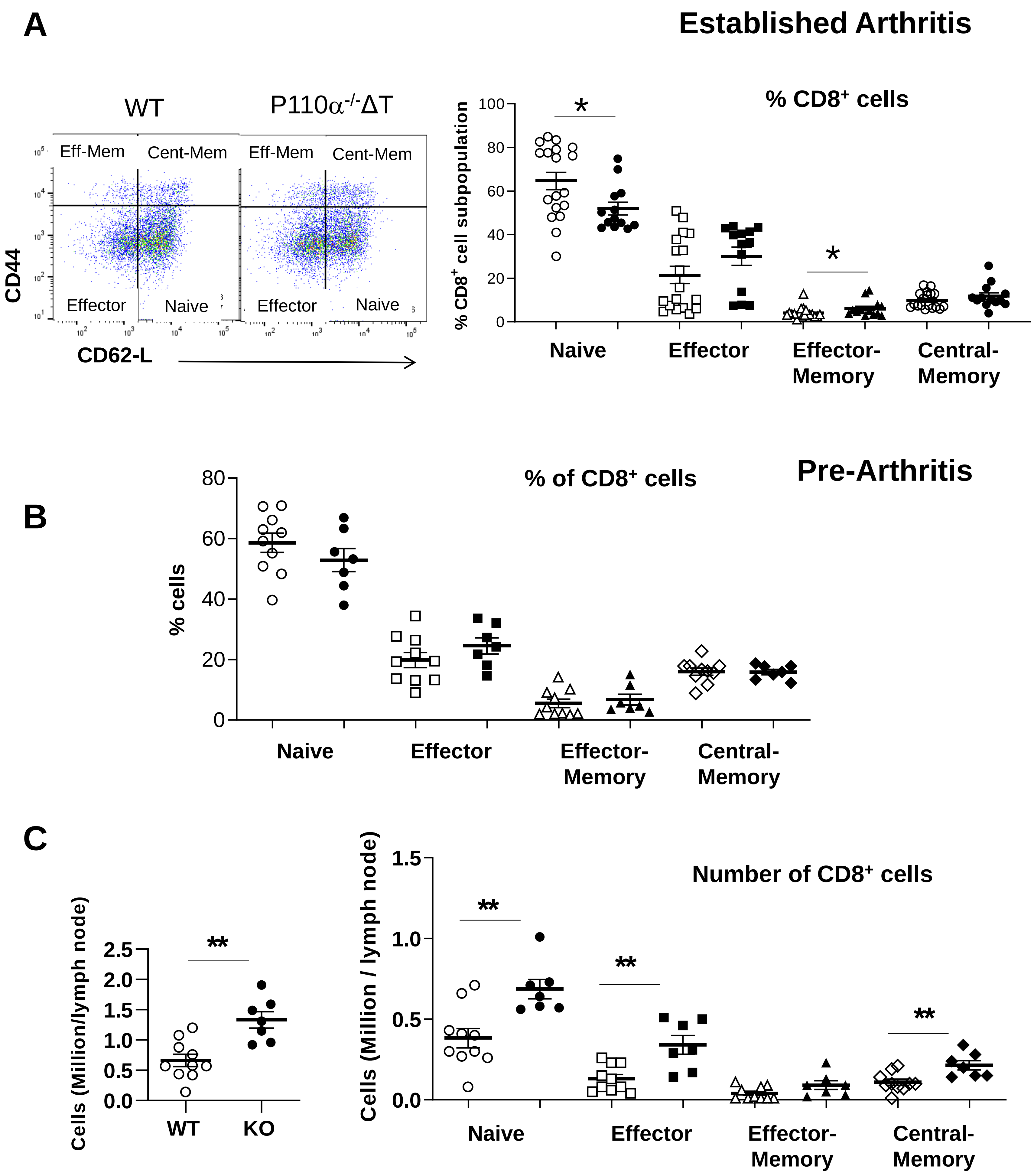

Figure S2. Analysis of CD8+ subpopulations in $p110\alpha^{-/-}\Delta T$ mice under **CIA.**

Upon long term experiments termination (established arthritis) **(A)** the fraction of $CD8^+$ cells in the lymph nodes of WT and $p110\alpha^{-/-}\Delta T$ mice was analyzed by cytometry. **(Left)** Representative dot-plots showing the expression of CD44 and CD62-L in gated $CD8^+$ T cells and the distribution of naive, effector and memory subpopulations **(Right)** Percentages of that subpopulations are represented.

Thirteen days **(B and C)** after Ag immunization, the fraction of $CD8^+$ cells subpopulations in the lymph nodes of WT and $p110\alpha^{-/-}\Delta T$ mice were analyzed. Percentage **(B)** and absolute numbers **(C)** of that subpopulations are represented.

N=23 for WT and n= 20 for $p110\alpha^{-/-}\Delta T$ mice in **A**, and N= 18 for WT and n=13 for $p110\alpha^{-/-}\Delta T$ mice in **B**.

*, $p<0.05$; **, $p<0.01$. Data show the mean ± SEM from individual mice analyzed.
